# Study on Periodic Pulsation Characteristics of Corn Grain in a Grain Cylinder during the Unloading Stage

**DOI:** 10.3390/foods10102314

**Published:** 2021-09-29

**Authors:** Han Tang, Changsu Xu, Xin Qi, Ziming Wang, Jinfeng Wang, Wenqi Zhou, Qi Wang, Jinwu Wang

**Affiliations:** College of Engineering, Northeast Agricultural University, Harbin 150030, China; tanghan@neau.edu.cn (H.T.); ChangsuXu@neau.edu.cn (C.X.); XinQi@neau.edu.cn (X.Q.); WangZiming@neau.edu.cn (Z.W.); jinfeng_w@126.com (J.W.); zwq@neau.edu.cn (W.Z.); wangqi@neau.edu.cn (Q.W.)

**Keywords:** corn grain, grain cylinder, unloading, periodic pulsation, discrete element method

## Abstract

The fluctuation effect of corn grain often occurs during the unloading stage. To accurately explore the periodic pulsation characteristics of corn grain during the unloading stage, a discrete model of corn grain was established, and the effectiveness of the discrete element method in simulating the corn grain unloading stage was verified by a 3D laser scanner and the “spherical particle filling method”. The grain cylinder was divided into six areas, and the periodic pulsation characteristics at different heights were explored through simulation tests. The results showed that the faster the average speed of corn grain changes in unit time, the more significant the periodic pulsation characteristics were as the height of grain unloading increased. The corn grain pulsateon in the grain cylinder exhibited gradual upward transmission and gradual amplification in the process of transmission. The average velocity decreased with increasing height. The direct cause of pulsation was the variation in the average stress between grain layers. Simulation analysis of grain unloading for different half cone angles of the grain cylinder was carried out. The change in corn grain average velocity over time in the area below 20 mm of the upper free surface was extracted. The results showed that the speed of the top corn grain increased with increasing the half cone angle, and the periodic pulsation phenomenon became more obvious with increasing the half cone angle at half cone angles of 30–65°. A half cone angle of 65–70° marked the critical state of corn grain flow changing from funnel flow to overall flow in the grain cylinder. This study provides a method for studying the periodic pulsation characteristics of different crops during the grain unloading stage and provides a technical reference for the safe design of grain unloading equipment.

## 1. Introduction

Corn is one of the most widely distributed crops in the world. There are a large number of planting areas from north latitude 58° to south latitude 40°. Corn plays an important role in ensuring world food security and economic stability [1,2]. As the main equipment of corn grain storage after harvest, grain cylinders can effectively extend the shelf life of corn and ensure its quality and safety. These cylinders are widely used in agricultural engineering, food engineering and animal husbandry engineering [3,4,5]. The flow of corn grain is a typical problem of bulk solid flow in the process of grain unloading. Velocity fluctuation and contact force disappearance occur in the grain cylinder. The dynamic effect of “granary vibration” and “granary music” is produced and can even lead to the failure of the structure [6,7,8].

Mechanical problems of bulk solids easily occur under the interaction of multiple factors when the vertical grain cylinder unloads grain by gravity, which can lead to granary destruction [9]. From the point of micromechanical analysis, the friction between grain particles and grain particles and between grain and silo walls are important factors for grain arching, representing the ultimate destruction mode [10]. Ayuga et al. [11] studied the effects of the grain dilatancy angle and the friction coefficient on the lateral pressure of silo walls. The effect of particles on the lateral pressure of the silo wall was found to be significantly different with different unloading heights. Yuan et al. [12], based on unloading grain model tests in an indoor grain cylinder, explored the mechanism of the overpressure phenomenon from the aspect of the flow state when unloading grain in the center of a grain cylinder. There are two flow forms in the unloading process: integral flow and funnel flow. It is easy to produce substantial overpressure in the mixed area of the two forms of flow [13,14]. Wang et al. [15] studied the static lateral pressure on the wall of a wheat grain cylinder under the state of grain storage and the dynamic lateral pressure on the wall of a wheat grain cylinder under the condition of central grain unloading. When the initial capacity of the grain cylinder was smaller, the deviation between the measured value of the static lateral pressure of the grain cylinder wall and the calculated value of the static lateral pressure of the national codes was greater, and the position of tubular flow on the upper surface of the grain was lower. The pressure of particles on the grain cylinder was not limited to the wall of the grain cylinder. Chen et al. [16] found that the pressure distribution at the bottom of the grain cylinder was more uniform when the accumulation height of wheat was low. The higher the accumulation height was, the more significant the annular distribution of bottom pressure was.

The development of computer technology allows for the accurate establishment of a grain particle model, providing an effective way to quantitatively analyze the interaction between grain particles and the grain cylinder wall. Xu et al. [17] simulated the whole unloading process of non-adhesive soft particles and adhesive hard particles in flat-bottomed grain cylinders using the discrete element method. The material modulus of particles had little effect on the unloading characteristics, but the adhesion of the particle surface had a significant hysteretic effect on the discharge flow rate. To this end, Feng et al. [18] added micro-particles with a large viscosity coefficient to simulate dust on the basis of the PFC discrete element program to accurately explore the meso-mechanism of grain unloading in grain cylinders. This provides some guidance for the study of the flow characteristics of bulk particles and the interaction mechanism between the bulk and the wall of grain cylinders. Wang et al. [19] studied the dynamic characteristics of the materials of grain cylinders and the interaction between the materials and cylinder walls using the finite element method. Related models were established, and good simulation results were obtained.

Resonance is also an important factor that can potentially lead to grain cylinder destruction, as the natural frequency of the structure is coupled with the pulsating frequency of particles [20]. Bartilson et al. [21] studied the natural frequency and mode sensitivity of symmetrical structures, providing a method for solving the natural frequencies of different structures of grain cylinders. From the analysis of particles, Muite et al. [22] carried out unloading experiments with different granular materials in grain cylinders with different natural frequencies. The results showed that the properties of the particle materials and the structure of the grain cylinder had a significant influence on the particle pulsation frequency. Tejchman et al. [23] pointed out that environmental factors such as temperature, humidity and electrostatic characteristics of different particles also have an influence on the pulsation frequency of particles.

The above studies focused mainly on the influencing factors and mechanisms of particles on the pressure of grain cylinder walls. However, the partial and overall relations of the pulsation characteristics between particles and between different heights in the grain cylinder and the influence of the structure of the grain cylinder on the pulsation characteristics have not been established. Discrete element simulation can more objectively reflect the micromechanical mechanisms of grain bulk in the grain cylinder. Therefore, it is of great practical significance to carry out research on this topic. In view of this, a discrete element model of corn grain was established in the present study. The grain unloading experiments were simulated based on the discrete element method in a vertical grain cylinder. The purpose of this paper is to study the variation in grain velocity pulsation and stress at different heights. The mechanism of particle pulsation was explored. The correlation of and difference in grain pulsation characteristics were analyzed in different areas of the grain cylinder. The effect of the half cone angle of the grain cylinder on the grain pulsation characteristics was explored. The relationship between the whole grain system and part of the grain system was revealed. This study provides a method and guidance for the safe design of grain unloading equipment.

## 2. Materials and Methods

### 2.1. Establishment of a Discrete Element Model for Corn Grain and Grain Cylinders

**Figure 1:**
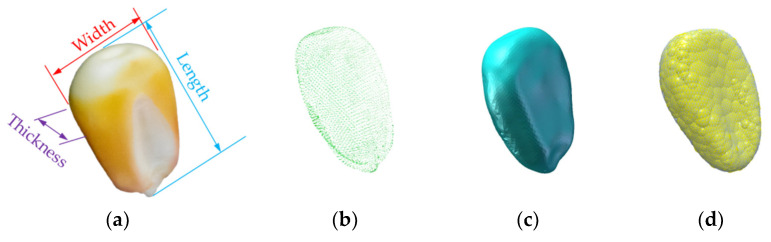
The corn grain of “Demeiya No. 1”: (**a**) corn grain prototype; (**b**) point cloud map; (**c**) 3D scanning model; (**d**) discrete element model.

The prototype of corn grain used in this study was “Demeia No. 1”, which is widely planted in Northeast China, and its shape was horse-toothed. The average values of the triaxial dimensions (length, width and thickness) were 12.35, 9.24 and 4.70 mm, respectively, as shown in Figure 1a. The overall structure of corn grain was complex. To establish the corn grain model accurately, a Reeyee X5 3D laser scanner (Nanjing Weibu 3D Technology Co., Ltd., Nanjing, China) was used to extract the three-dimensional geometric feature parameters of corn grain. A 3D laser scanner projected gratings onto the surface of the corn grain. According to the shape of the fringes changing with the curvature, the spatial coordinates of each point of the surface were accurately calculated by using the orientation method and the triangulation method. 3D point cloud data were generated [24], as shown in Figure 1b. Shading, denoising, point cloud registration, point cloud triangulation, merging and model correction were carried out in turn. Using automated reverse engineering software to convert scan data into an accurate digital model [25], the ideal 3D scanning model of corn grain was obtained, as shown in Figure 1c. This 3D laser-scanned corn grain model was imported into EDEM and filled with spherical element particle polymerization. The discrete element model of corn grain is shown in Figure 1d.

The grain cylinder was divided mainly into shallow and vertical grain cylinders. Vertical grain cylinders have the advantages of easy storage and release of grains and large storage capacity and are widely used in large farms and grain storage enterprises [26]. The vertical grain cylinder is composed mainly of a cylindrical grain storage cylinder and hopper. The ratio of height to diameter of the cylindrical grain storage cylinder is called the height-diameter ratio. Vertical grain cylinders with a height-diameter ratio greater than 1.5 are more susceptible to particle pulsation [27]. To clearly observe the pulsation characteristics between grain flows, the effect of the vertical grain cylinder material on the corn grain was not considered. Transparent plexiglass was used to make vertical grain cylinders in this study. There is no strict standard for the specific height and diameter of vertical grain cylinders in actual production. A vertical grain cylinder with a common height of 30 m and a diameter of 14 m was taken as the research prototype. The model of the vertical grain cylinder was designed according to similarity theory.

Similarity theory incorporates the geometric similarity between the model and the prototype and requires that the ratio of the corresponding linear dimensions is the same and that the corresponding angles are equal [28]. The calculation equation is as follows:

(1) $$\frac{H}{H_{M}}=\frac{D}{D_{M}}=\zeta$$

where H is the height of the vertical grain cylinder model, mm; $H_{M}$ is the height of the vertical grain cylinder prototype, mm; D is the diameter of the vertical grain cylinder model, mm; $D_{M}$ is the diameter of the vertical grain cylinder prototype, mm; and ζ is the scaling coefficient. The model was designed with a height of 300 mm and a diameter of 140 mm, and the scaling coefficient ζ is 0.01.

(2) $$\alpha =\alpha_{M}$$

where α is the half cone angle of the hopper for the vertical grain cylinder model (°) and $\alpha_{M}$ is the half cone angle of the hopper for the vertical grain cylinder prototype (°).

Similarity theory requires that the motion of the model be similar to the prototype. The velocity ratio of the medium passes equally through the corresponding point between the model and the prototype, and the direction of the velocity vector is the same. To accurately simulate the speed of the corn grain passing through a certain point in the grain cylinder prototype, Equation (3) was established.

(3) $$\frac{v_{1}}{v_{1M}}=\frac{v_{2}}{v_{2M}}=\zeta$$

where $v_{1}$ is the speed of the “1” point for the vertical grain cylinder model, mm/s; $v_{1M}$ is the speed of the “1” point for the vertical grain cylinder prototype, mm/s; $v_{2}$ is the speed of the “2” point for the vertical grain cylinder model, mm/s; and $v_{2M}$ is the speed of the “2” point for the vertical grain cylinder prototype, mm/s. The speed of corn grain passing through a certain point or area of the vertical grain cylinder model calculated in the simulation was $\frac{1}{\zeta }$ as fast as in the vertical grain cylinder prototype.

If $u_{i}$ represents the velocity of the *i*-th particle in the cylindrical grain storage cylinder along the direction of gravity, the average velocity of all particles $\bar{u}$ in the cylindrical grain storage cylinder at that time is as follows:

(4) $$\bar{u}=\frac{1}{n}\sum_{1}^{n}u_{i}$$

where *n* is the total number of grain particles.

The average velocity of particles $\bar{u^{’}}$ in a cylindrical grain storage cylinder over a certain period of time is as follows:

(5) $$\bar{u^{’}}=\frac{1}{t}\sum_{0}^{t}\bar{u}$$

where *t* is the total sampling time, s; The equation discretizes the time, and the fixed time step is 5% of the Rayleigh time step.

The fluctuation degree of the average velocity was quantitatively evaluated by the standard deviation σ to characterize the pulsation characteristics of the particles. The standard deviation of the average speed is as follows:

(6) $$\sigma =\sqrt{\sum_{j=1}^{t}\frac{{\left({\bar{u}}_{j}-\bar{u^{’}}\right)}^{2}}{t-1}}$$

where ${\bar{u}}_{j}$ is the average speed at the time of grain unloading j, mm/s.

**Figure 2:**
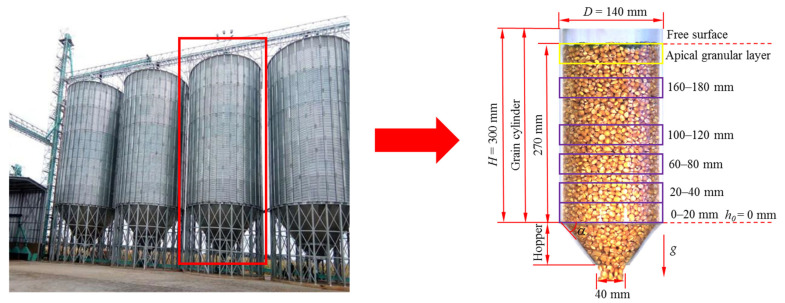
Structure diagram of vertical grain cylinder.

To explore the effects of the half cone angle of the hopper and different heights of stored grain on the pulsation characteristics of grain flows, the half cone angle was set to 30°, 35°, 40°, 45°, 50°, 55° and 60°. The grain storage was divided into five fixed areas by taking the junction of the cylindrical grain storage cylinder and the hopper as the zero point. The five fixed areas were 0–20, 20–40, 60–80, 100–120 and 160–180 mm. The top corn grains were selected for the range below 20 mm on the upper free surface. There were six analysis areas as shown in Figure 2.

**Figure 3:**
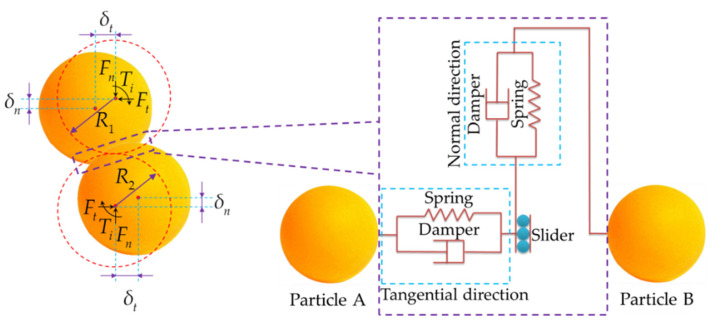
Mechanical contact model: The red dotted lines show the positions of particles A and B without considering the force; yellow particles represent the state after contact.

The grain moisture content of corn grain stored in grain cylinders is lower. Corn grain can be idealized as a nonviscous body [29]. The Hertz–Mindlin (no slip) model was used as the contact mechanics model to establish the discrete element model fo grain–grain and grain–plexiglass [30,31]. Changes in parameters such as the force, displacement and velocity of the corn grain in the process of motion were all considered to be caused by the deformation produced by the collision. The contact force was decomposed into tangential force $F_{t}$ and normal force $F_{n}$. The mechanical contact model was established as shown in Figure 3.

When particle A and particle B are in elastic contact, the calculation formula of normal overlap $\delta_{n}$ is as follows [32]:

(7) $$\delta_{n}=R_{1}+R_{2}-\left|r_{1}-r_{2}\right|$$

where *R*_1_ and *R*_2_ are the radii of particle A and particle B, respectively, mm; $r_{1}$ and $r_{2}$ are the spherical center position vectors of particle A and particle B, respectively, mm.

The contact surface between two spherical particles is circular. The contact radius *a* is as follows:

(8) $$a=\sqrt{\delta_{n}R^{*}}$$

(9) $$\frac{1}{R^{*}}=\frac{1}{R_{1}}+\frac{1}{R_{2}}$$

where $R^{*}$ is the equivalent radius between particles A and B, mm.

The normal force of contact between particles A and B is as follows:

(10) $$F_{n}=\frac{4}{3}E^{*}\sqrt{R^{*}}\delta_{n}{}^{\frac{2}{3}}$$

where $F_{n}$ is the contact normal force, N; $E^{*}$ is the equivalent modulus of elasticity, MPa.

(11) $$\frac{1}{E^{*}}=\frac{1-v_{1}{}^{2}}{E_{1}}+\frac{1-v_{2}{}^{2}}{E_{2}}$$

where $v_{1}$ is Poisson’s ratio of particle A; $v_{2}$ is Poisson’s ratio of particle B; $E_{1}$ is the elastic modulus of particle A, MPa; and $E_{2}$ is the elastic modulus of particle B, MPa.

The equation of the normal damping force for particles A and B is as follows:

(12) $$F_{n}{}^{d}=-2\sqrt{\frac{5}{6}}\beta \sqrt{S_{n}mv_{n}{}^{vel}}$$

where m is the equivalent mass; g, $m={\left(\frac{1}{m_{1}}+\frac{1}{m_{2}}\right)}^{-1}$, $m_{1}$ and $m_{2}$ are the weights of particles A and B, respectively; $v_{n}{}^{vel}$ is the normal component of the relative velocity, m/s; β is the coefficient related to the recovery coefficient *e*; and $S_{n}$ is the normal stiffness.

The calculation equations are as follows:

(13) $$\beta =\frac{\ln e}{\sqrt{\ln^{2}e+\pi^{2}}}$$

(14) $$S_{n}=2E^{*}\sqrt{R^{*}\delta_{n}}$$

The contact tangential force between particles A and B is as follows [33]:

(15) $$F_{t}=-S_{t}\delta_{t}$$

where $F_{t}$ is the contact tangential force, N; $\delta_{t}$ is the tangential overlap.

(16) $$S_{t}=8G^{*}\sqrt{R^{*}\delta_{n}}$$

where $S_{t}$ is the tangential stiffness and $G^{*}$ is the equivalent shear modulus.

(17) $$G^{*}=\frac{2-v_{1}{}^{2}}{G_{1}}+\frac{2-v_{2}{}^{2}}{G_{2}}$$

where $G_{1}$ is the shear modulus of particle A and $G_{2}$ is the shear modulus of particle B.

The tangential damping force between particles A and B is as follows:

(18) $$F_{t}{}^{d}=-2\sqrt{\frac{5}{6}}\beta \sqrt{S_{t}mv_{t}{}^{vel}}$$

where $F_{t}{}^{d}$ is the tangential damping force, N; $v_{t}{}^{vel}$ is the tangential component of the relative velocity, m/s.

The friction f between particles A and B is as follows:

(19) $$f=\mu_{s}F_{n}$$

where $\mu_{s}$ is the static friction coefficient.

The rolling friction between particles A and B is shown by applying a moment $T_{i}$ on the contact surface.

(20) $$T_{i}=-\mu_{r}F_{n}R_{i}\omega_{i}$$

where $\mu_{r}$ is the rolling friction coefficient; $R_{i}$ is the distance from the centroid of the particle to the contact point, mm; and $\omega_{i}$ is the unit angular velocity vector of the particle at the contact point, rad/s.

According to the study on the determination of corn grain material characteristics in the early stage and related references, simulation parameters were set by EDEM simulation, as shown in Table 1.

With reference to the coordinates of the model, the z-axis direction was selected as the gravity direction. Gravity acceleration was set to −9.81 m/s^2^. Corn grains were modeled as accumulating naturally in the grain cylinder by using the “falling rain method” [38]. The initial speed was zero. When the grain storage height of 270 mm was reached, the total number of particles was 8264 (taking the half cone angle of 45° as an example, there was a certain difference in the total number of particles due to the difference in the half cone angle). The grains settled naturally, and then the discharge port was opened to start the simulation after standing for 1 s. The total simulation time of each group was set to 15 s. The Rayleigh time step was automatically calculated by the program according to the set particle radius, density and other parameters. We set the fixed time step between two iterative calculations as 5% of the Rayleigh time step, so as to shorten the discrete time interval and ensure the comprehensiveness of the data.

**Figure 4:**
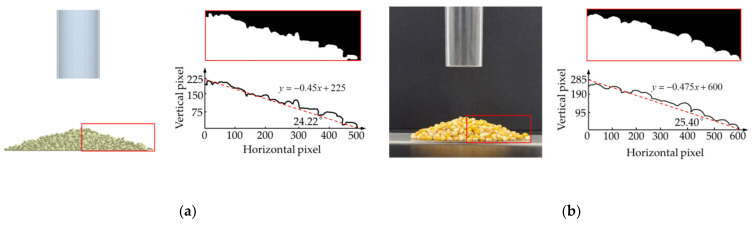
Comparative determination of natural rest angle of corn grain by simulation and experiment: (**a**) EDEM simulation results; (**b**) bench test results.

To verify the accuracy of the discrete element model of corn grain before the simulation, EDEM simulations and bench tests were used to compare and measure the natural repose angle. The cylinder was placed vertically on the plane (the cylinder and the plane were plexiglass material). The diameter of the cylinder was 4–5 times the maximum grain size. The ratio of height to cylinder diameter was 3:1 [39,40]. A certain number of corn grains were placed in the cylinder. The cylinder was perpendicular to the plane and rose slowly at a speed of 0.3 m/s. The points when corn grain stopped accumulating, slipping and rolling were determined. Consistency between the EDEM simulation and bench test was ensured. The tests were repeated three times, and the results were averaged. To accurately determine the natural repose angle of corn grains, the accumulation of the corn population was photographed in three-dimensional space. Using MATLAB software to process image noise, grayscale and binarization, the unilateral contour curve of corn grain accumulation was extracted. The result is shown in Figure 4. The average natural repose angle measured by EDEM simulation was 24.22°. The average natural repose angle measured by the bench test was 25.40°. The relative error was 4.65%. The main reason was that the discrete element model of corn grain was formed by the aggregation of small spheres. The surface area of corn grain was increased. The friction force of the contact parts of the adjacent grains also increased accordingly in the process of free accumulation. This led to a decrease in corn grain fluidity. The overall difference in corn grain shapes in the bench test was a source of error. The corn grain discrete element model was accurate and effective within the allowable error. The results lay a foundation for the study of the periodic pulsation characteristics of corn grains in grain cylinders.

### 2.2. Comparative Analysis of Simulation and High-Speed Camera Test

**Figure 5:**
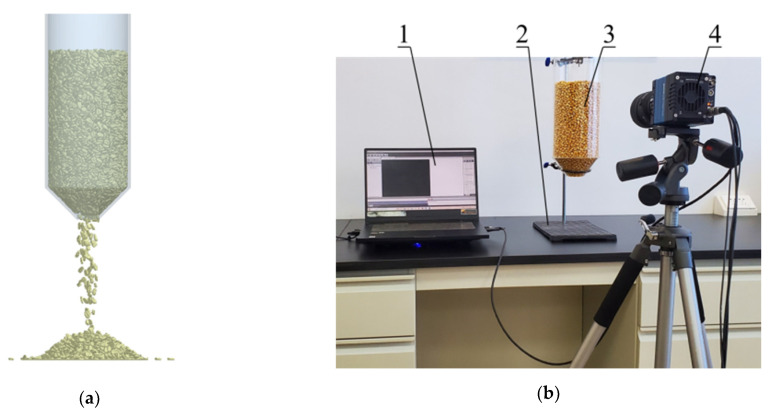
Simulation of corn grain flow and high-speed camera test: (**a**) EDEM simulation; (**b**) high-speed camera test. 1. PC; 2. Bench; 3. Vertical grain cylinder model; 4. High-speed camera.

To check the consistency between the simulation and bench test of corn grain in the vertical grain cylinder model and to verify the accuracy of the simulation analysis of grain pulsation characteristics in grain cylinders, a comparative study on corn grain flow simulation and a high-speed camera test were carried out, as shown in Figure 5. The corn grain height of the vertical grain cylinder model in the simulation and high-speed camera test was set to 270 mm. A half cone angle of 45° was taken as an example. The junction of the cylindrical grain storage cylinder and hopper was taken as the zero point to explore the relationship between the free surface height and average velocity over time.

**Figure 6:**
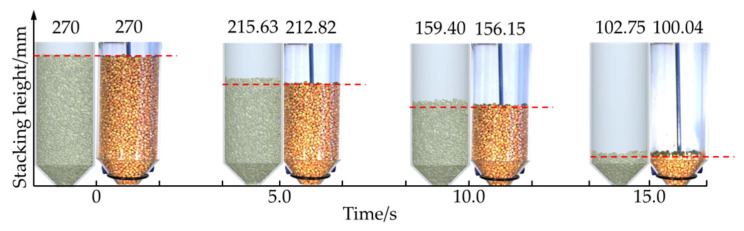
Comparative results of the corn grain flow simulation and high-speed camera test.

The high-speed camera test instruments mainly included a PC, bench, vertical grain cylinder model and high-speed camera. Among them, the high-speed camera (Dimax cs model) was produced by the German Company PCO (Bayern, Germany). The resolution was 1920 × 1080. The spectral range was 290–110 nm. The maximum frame rate reached 2128 fps. The vertical grain cylinder model was adjusted to the vertical direction, and the frame number of the high-speed camera was set to 1000 fps before the start of the test. The high-speed camera was started, and the hopper opening was opened. The data stored in the high-speed camera were transmitted to the PC through the network cable connected to the PC for preservation. The tests were repeated three times, and the results were averaged. The comparison results of the corn grain flow simulation and high-speed camera test are shown in Figure 6.

The accumulation height of corn grain in the EDEM simulation and high-speed camera test was 270 mm at 0 s from the analysis of Figure 6. The corn grains fell to a certain height as the test progressed. However, the falling speed of corn grain in the EDEM simulation was slower than in the high-speed camera test. It was verified that the fluidity of the corn grain model established in the EDEM simulation was poor. The maximum error of the comparison between the two was 2.71% in the process of falling. The model of corn grain and vertical grain cylinder was established within the allowable range of error, and the simulation parameters were accurate and reliable.

## 3. Results and Discussion

### 3.1. Pulsation Characteristics of Average Velocity for Corn Grain at Different Storage Heights

**Figure 7:**
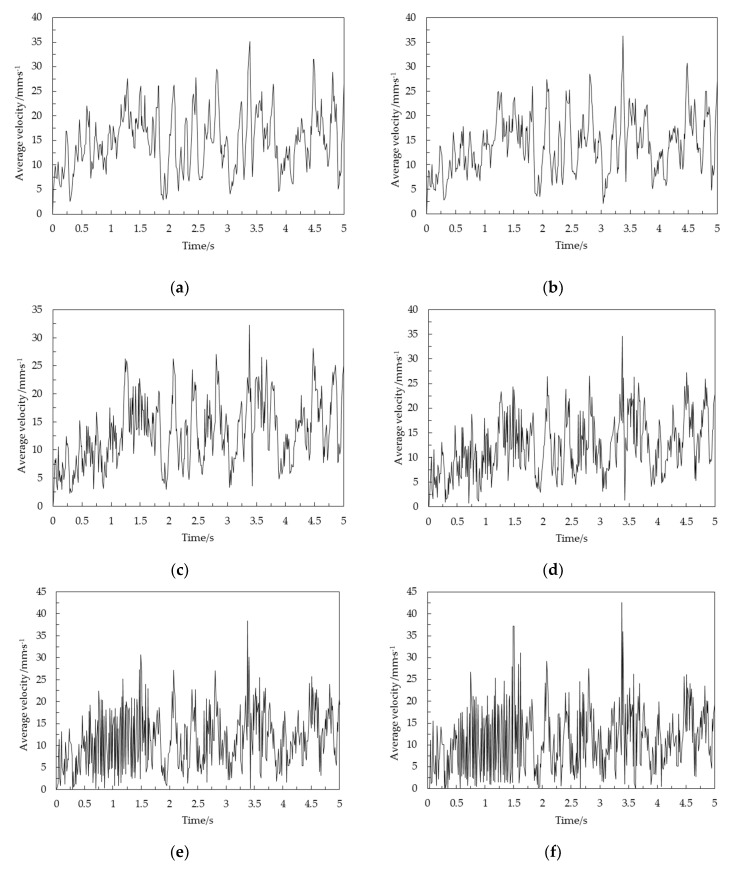
Average velocity over time of the corn grain in different areas: (**a**) average velocity over time of the corn grain in the 0–20 mm area; (**b**) average velocity over time of the corn grain in the 20–40 mm area; (**c**) average velocity over time of the corn grain in the 60–80 mm area; (**d**) average velocity over time of the corn grain in the 100–120 mm area; (**e**) average velocity over time of the corn grain in 160–180 mm area; (**f**) average velocity over time of the corn grain in the area below 20 mm on the upper free surface.

**Figure 8:**
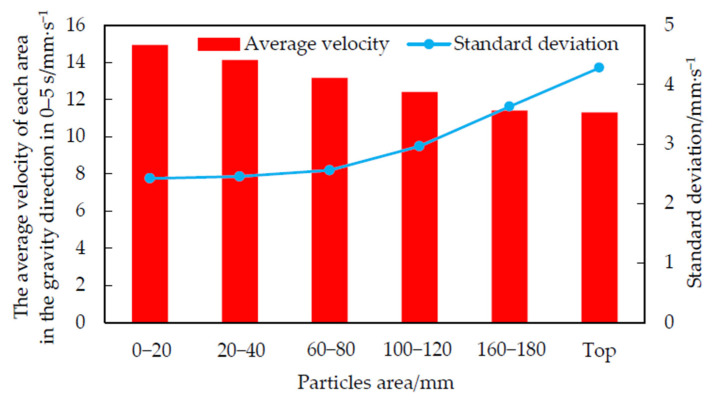
Average velocity and standard deviation of corn grain in each area over 0–5 s.

**Figure 9:**
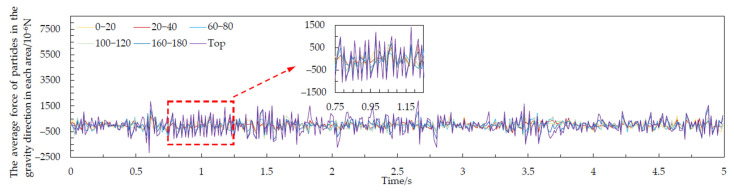
Average force of the corn grain in different areas over 0–5 s.

**Figure 10:**
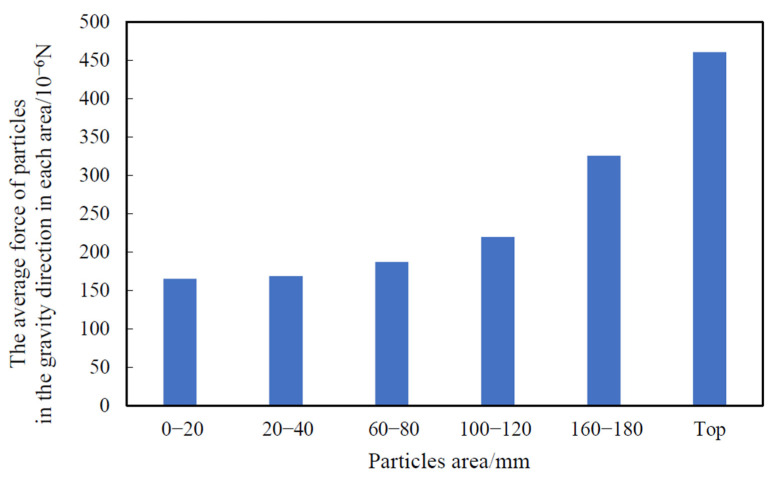
Average force of corn grain in each area over 0–5 s (bar chart after taking absolute value).

**Figure 11:**
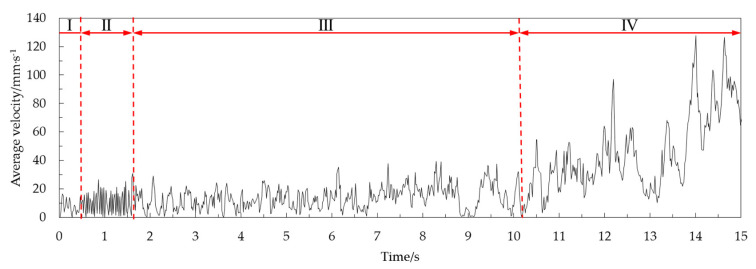
Average velocity of the corn grain in the area below 20 mm on the free surface at a half cone angle of 45°.

**Figure 12:**
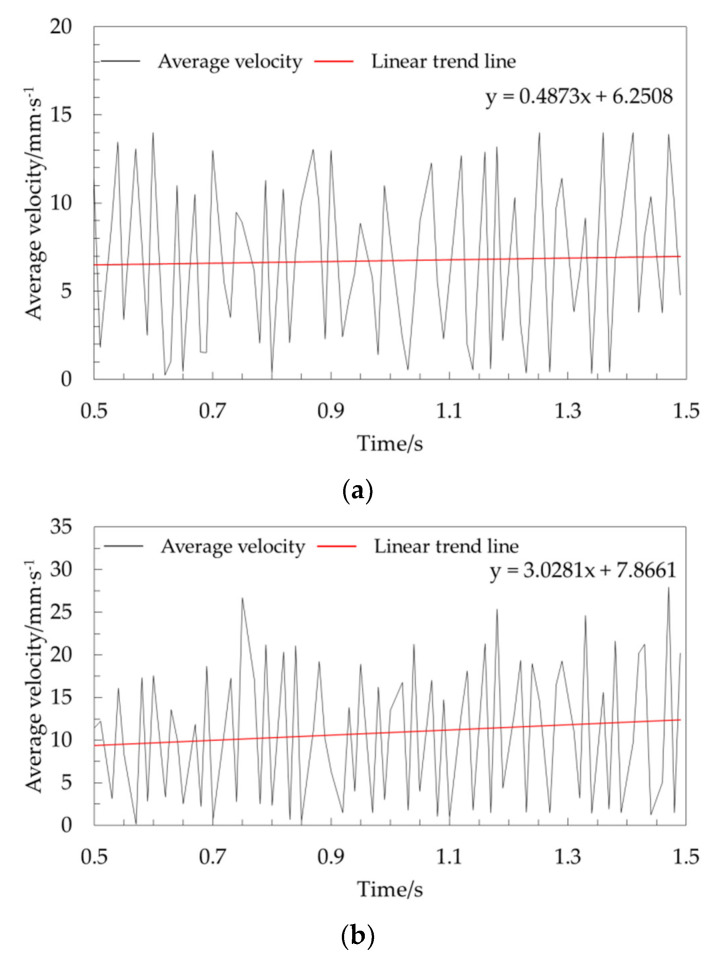

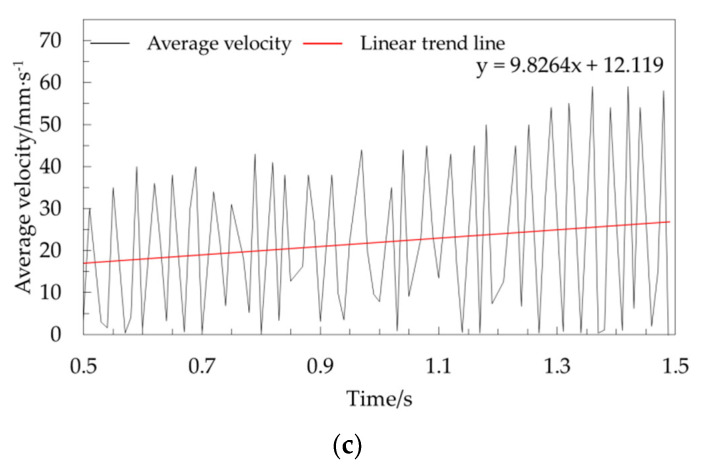
Average velocity in 1 s during the period of periodic pulsation at different half cone angles below 20 mm on the free surface: (**a**) average velocity over 1 s during the period of periodic pulsation at a half cone angle of 30° below 20 mm on the free surface; (**b**) average velocity over 1 s during the period of periodic pulsation at a half cone angle of 45° below 20 mm on the free surface; (**c**) average velocity over 1 s during the period of periodic pulsation at a half cone angle of 60° below 20 mm on the free surface.

To reveal the longitudinal pulsation characteristics of corn grains in a vertical grain cylinder, a half cone angle of 45° was taken as an example. The average velocities of five fixed areas (0–20, 20–40, 60–80, 100–120 and 160–180 mm) and the areas below 20 mm of the upper free surface were analyzed according to Equation (4), as shown in Figure 7, Figure 8, Figure 9, Figure 10, Figure 11 and Figure 12. The total extraction time was 5 s. The height of corn grain in the cylindrical grain storage cylinder decreased to 215.63 mm during this time. This study focused mainly on the periodic pulsation period to characterize the pulsation characteristics of corn grains in different areas and the relationship between grains.

Figure 7a shows the variation curve of the average velocity over time for corn grain in the 0–20 mm area after opening the grain unloading port. The average speed of the corn grain gradually increased. Stable fluctuations began to occur upon reaching 15 mm/s. The range of speed fluctuation was approximately 4–35 mm/s. A large number of double peaks and multiple peaks occurred at the same time. The change in grain speed exhibited irregular fluctuations. There was no stable fluctuation cycle.

Figure 7b shows the variation curve of the average velocity over time for corn grain in the 20–40 mm area. The overall trend did not change much relative to the average speed of the corn grain in the 0–20 mm area. However, the speed fluctuation range decreased. The double peaks and multiple peaks were significantly reduced in the process of fluctuation. The change in grain speed exhibited irregular fluctuation. There was no stable fluctuation cycle.

Figure 7c shows the variation curve of the average velocity over time for corn grain in the 60–80 mm area. The amplitude of the waveform in this area became larger and was mainly concentrated at 3–27 mm/s. In addition, the waveform of average velocity changed faster per unit time. The double peaks and multiple peaks were significantly reduced in the process of fluctuation.

Figure 7d shows the variation curve of the average velocity over time for corn grain in the 100–120 mm area. The waveform of the amplitude center in this area generally showed a downward trend. The range of speed fluctuation was concentrated in 1–25 mm/s. The peaks and troughs were clearly discernible at this time.

Figure 7e shows the variation curve of the average velocity over time for corn grain in the 160–180 mm area. The average speed of corn grain fluctuated when it reached 5 mm/s in this area. The range of speed fluctuation was approximately 1–23 mm/s. The minimum average speed was 0 mm/s. The fluctuation amplitude of the average velocity was significantly higher than that in the 0–20 mm area at this time. The change speed of the average velocity waveform was significantly improved per unit time. The waveform began to show regular periodic fluctuations at this time. Double peaks and multiple peaks were almost nonexistent in the process of fluctuation.

Figure 7f shows the variation curve of the average velocity over time for corn grain in the area below 20 mm on the upper free surface. The range of average velocity fluctuation in this area was approximately 0–22 mm/s. There were no double peaks or multiple peaks in the fluctuation process. Regular periodic fluctuations were prominent in this area. The fluctuation range was approximately 0–22 mm/s and was mainly manifested in the period of 0.50–1.65 s. The peaks and troughs of other waveforms were clearly discernible. However, the regularity was poor. The phenomenon of periodic pulsation was not obvious. These effects marked the transition section of the regular waveform.

The above results show that the variation trend and waveform of the average velocity over time of the corn grain with different grain storage heights were significantly different. The closer the distance to the top of the cylindrical grain storage cylinder, the faster the average speed of corn grain changed per unit time, and the more prominent the characteristics of periodic pulsation. The corn grain at the bottom accelerated to a certain speed and then fluctuated irregularly. Its change typically appeared as a random process. For smaller heights, the minimum velocity variation of each particle layer fluctuated little. For the middle and high granular layer, with the increase of height, the minimum velocity of the granular layer gradually decreased and tended to be close to 0. However, it gradually showed notable characteristics of periodic pulsation (the peaks and troughs could be clearly identified, and there was a stable fluctuation period at the same time). The grain pulsation in the grain cylinder was gradually transmitted upward, and the periodic pulsation characteristics were gradually magnified in the process of transmission.

Figure 8 shows the average speed and standard deviation of corn grain in each area over 0–5 s. The closer the distance to the top of the cylindrical grain storage cylinder, the smaller the average velocity of the grain layer. The main reason was that the grains in the lower end of the cylindrical grain storage cylinder flowed out of the grain cylinder first during the grain unloading process. It was impossible for the upper grain to overtake the lower grain to flow out of the grain cylinder first with increasing height. Therefore, the average speed decreased with increasing height.

The standard deviation can not only reflect the magnitude of fluctuation amplitude, but also characterize the frequency difference of velocity fluctuation (for data points with the same number and amplitude, the greater the frequency, the greater the standard deviation) [27]. According to Figure 7 and Figure 8, with an increase in height, the amplitude was not significantly affected, and the standard deviation of fluctuation gradually increased, indicating that the fluctuation frequency gradually increased. Figure 8 verified the analysis of Figure 7.

The variation in the average force of the grain layer along the direction of gravity at each height was simulated by EDEM 18.0, as shown in Figure 9. The waveforms of the adjacent grain layers were similar, and the change periods were the same. Corn grains showed a periodic pulsation consistent with velocity pulsation in the process of grain unloading. The change in the average force between the grain layers was the direct cause of the pulsation phenomenon. Among them, the fluctuation range of stress on the top grain was the largest. We took the absolute value of the stress and averaged it in each area in the 0–5 s range. The force increased gradually with increasing height, as shown in Figure 10. This was mainly because the grain layer was subjected to the very large reaction force of the adjacent grain layer in the process of oscillation. The movement of the bottom grain was hindered by the adjacent grain layer. However, the grains at the top had no hindrance and a higher degree of freedom. Therefore, the top grain was subjected to a greater force in the direction of gravity. The phenomenon of periodic pulsation of velocity became increasingly prominent with increasing height. The energy of the underlying grain could not be fully reflected in its own movement from the view of energy and passed on to the adjacent grain layer. Finally, it was shown to a greater extent by the top grain layer.

There were significant differences in the movement between layers of corn grain accumulated in the cylindrical storage cylinder, including the amplitude of average velocity fluctuation, the speed of fluctuation per unit time and the significance of pulsation characteristics. The grain layers were related to each other, including similar pulsation waveforms and the same pulsation period. Corn grains not only were limited to the whole grain cylinder system but also had independent movement characteristics.

### 3.2. Effect of the Half Cone Angle on the Pulsation Characteristics of the Grain Layer

The above studies revealed the pulsation characteristics of the grain layer at different heights. The characteristics of periodic pulsation became more prominent with increasing grain layer height. To analyze the influence of different half cone angles on periodic pulsation characteristics, a simulation analysis of grain unloading was carried out in seven groups of different half cone angles. The variation in the average velocity over time for corn grains in the area below 20 mm on the upper free surface was extracted with half cone angles of 30°, 45° and 60°.

We considered a half cone angle of 45° as an example. The change in average velocity over time in the area below 20 mm on the free surface of the corn grain during the 15 s time period of grain unloading in the grain cylinder was extracted, as shown in Figure 11. The corn grain was in the fluctuating stage during the whole grain unloading stage. The period of 0–0.5 s marked the beginning stage of grain unloading. The speed fluctuation range was small at this time. It had a clearly visible single peak and did not have a periodic law. Phase I was labeled the fluctuation transition period. The peak and trough of the velocity curve in the period of 0.5–1.65 s were clearly discernible. There was a stable fluctuation cycle in this stage. Phase II was labeled the periodic pulsation period. The fluctuation of the average velocity curve in the period of 1.65–10.20 s was small. Double peaks and multiple peaks were more common at this stage. The fluctuation period was irregular. Phase III was labeled the irregular fluctuation period. The average velocity curve fluctuated greatly and had no periodic law in the period of 10.20–15 s. The overall average speed showed an upward trend at this stage. Phase IV was labeled the volatility jump period.

To perform an in-depth analysis of the influence of different half cone angles on the characteristics of periodic pulsation, the curves of the periodic pulsation period within 1 s (Phase II) were extracted and compared and analyzed, as shown in Figure 12.

When the half cone angle was 30°, the linear trend of the average velocity in 1 s during the periodic pulsation period was at the basic level, as shown in Figure 12. The results showed that when the half cone angle was small in the unloading stage, the average grain velocity of corn was relatively stable over time. With an increase in the half cone angle, the average velocity of the grain increased gradually. The linear trend of the average velocity in 1 s during the periodic pulsation period increased with an increasing half cone angle, as did the corresponding amplitude. The phenomenon of periodic pulsation of the top grains became more significant with the increasing half cone angle in the range of 30–60°.

To quantitatively evaluate the difference in velocity fluctuation in periodic pulsation periods with different half cone angles, the standard deviation of the velocity for seven groups of simulation experiments within 1 s in the periodic pulsation period was calculated. However, the linear trend line in Figure 12 showed that the speed increases gradually with time, which did not meet the definition of standard deviation. Therefore, the central difference method [41] was used to deal with the original data, and then the standard deviation was solved. The first-order central difference equation is as follows:

(21) $$\Delta V_{t}=\frac{1}{2}(V_{t+1}-V_{t-1})$$

where $\Delta V_{t}$ is the average velocity after the difference at time *t*, mm/s; $V_{t+1}$ is the original average speed at time *t* + 1, mm/s; and $V_{t-1}$ is the original average speed at time *t* − 1, mm/s.

**Figure 13:**
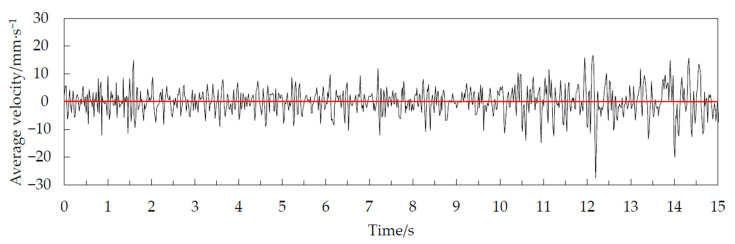
Average velocity of the corn grain in the area below 20 mm on the free surface at a half cone angle of 45° using the central difference method.

**Figure 14:**
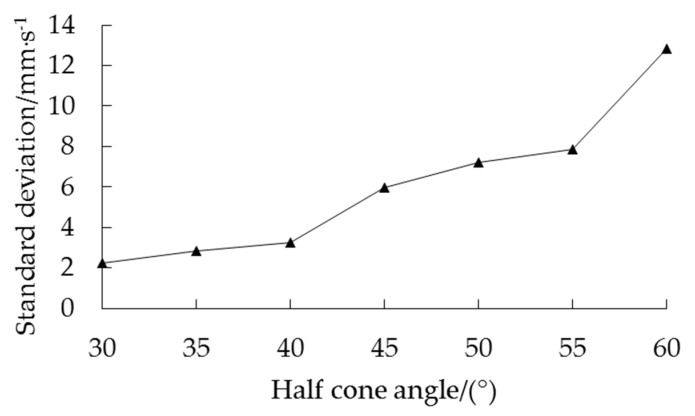
Standard deviation of the average velocity in 1 s in the periodic pulsation period with a half cone angle in the range of 30–60°.

After using central difference processing, the data basically fluctuated around the zero-scale line, and the average value remained the same. The speed fluctuation period was not changed. The amplitude of the data with large fluctuations remained large after processing. This was more conducive to clearly observing the pulsating characteristics of the average speed for corn grains. The average velocity of corn grains in the area below 20 mm on the free surface was treated by the central difference when the half cone angle was 45°, as shown in Figure 13. Finally, the speed standard deviation within 1 s in the periodic pulsation period for 7 groups of simulation experiments was obtained, as shown in Figure 14.

The standard deviation increased with increasing half cone angle, as shown in Figure 14. The speed of the top corn grain tended to become unstable with increasing half cone angle during grain unloading. The main reason was that the amplitude of periodic severe pulsation increased with increasing half cone angle. The corn grain pulsation at the top of the grain cylinder increased with increasing half cone angle in the range of 30–60°. It was verified that the linear trend of the average velocity within 1 s in the periodic pulsation period increased with increasing half cone angle.

### 3.3. Discussion

Loose corn grains accumulate in the natural state. The principle of designing the shape and structure of the grain cylinder is to adapt to the flow properties of loose materials and to obtain the overall flow of materials [42]. The unloading rate of the overall flow is stable, and the unloading density is uniform. The order of grain unloading is first in, first out [43]. When the corn grain flows downward in the grain cylinder, there is friction between the corn grain and the wall of the grain cylinder. When the friction force in the vertical direction is less than the gravity of the corn grain, the grain can flow smoothly [44]. The friction force caused by the lateral pressure of the corn grain against the grain cylinder wall is much less than the gravity of the corn grain in the cylindrical grain storage cylinder section. The fluidity of the grain is not affected. To make the corn grain flow an overall flow in the grain cylinder, a reasonable half cone angle is selected in the hopper section. Normally, the larger the half cone angle is, the more favorable the grain flow is [45]. The necessary condition for the formation of overall flow is that the half cone angle of the hopper θ is greater than $\theta_{\max}$ [46].

(22) $$\theta_{\max}=90^{\circ }-(90^{\circ }+\emptyset -\delta -\arcsin(\sin\delta /\sin\emptyset ))/2$$

where ∅ is the internal friction angle, (°), and the internal friction angle is equal to the natural rest angle for bulk materials such as corn grains, ∅ = 24.22°; δ is the friction angle between corn grain and grain cylinder wall, (°), δ = 19.34°. In addition, θ > 69.47°.

**Figure 15:**
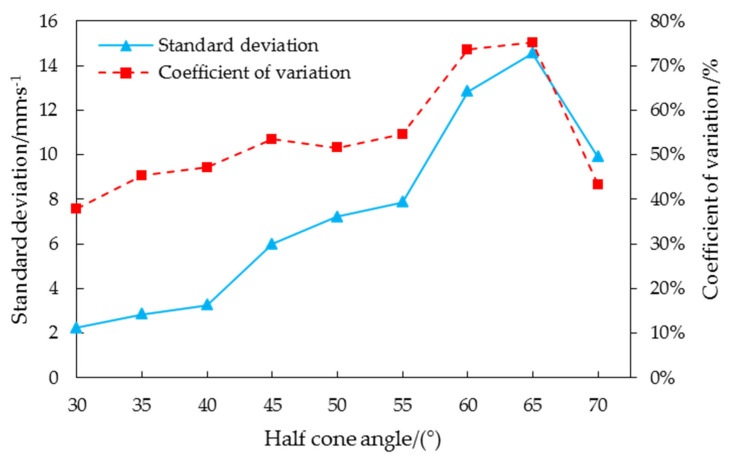
Standard deviation and coefficient of variation of the average velocity in 1 s in the periodic pulsation period with the half cone angle in the range of 30–70°.

The standard deviation and coefficient of variation of the average velocity in 1 s during the periodic pulsation period were further explored when the half cone angles were 65° and 70°, as shown in Figure 15.

As can be seen from Figure 15, the velocity standard deviation gradually increased in the range of 30–65° and gradually decreased in the range of 65–70°. In order to further explore the dispersion degree of average velocity under different half cone angles, the coefficient of variation was solved by Equation (23). It can be seen from Figure 15 that the coefficient variation of velocity increased in the range of 30–65° and suddenly decreased to a very small value in the range of 65–70°. The results showed that in the range of 30–65°, corn grains formed funnel flow in the grain cylinder, and the unloading velocity was unstable under the funnel flow state. In the range of 65–70°, corn grains form an overall flow in the grain cylinder, and the unloading velocity in the overall flow state was more stable than that in the funnel flow. It was verified that the half cone angle of corn grain from funnel flow to overall flow in the grain cylinder made of plexiglass material was between 65° and 70°.

(23) $$c_{v}=\frac{\sigma }{\bar{u^{’}}}\times 100\%$$

where $c_{v}$ is the coefficient of variation, %.

The pulsation of corn grain caused the vibration of the grain cylinder and led to the noise pollution of “grain cylinder music” [47,48], accompanied by substantial security risks. The greater the corn grain accumulation height in the grain cylinder was, the greater the probability of pulsation during grain unloading. To avoid the vibration of corn grains and grain cylinders, the height of corn grain accumulation in the grain cylinder should be moderate, and the half cone angle of the hopper is expected to make the material flow in the grain cylinder as an overall flow as made possible in the study of this paper. Poor grain unloading and large speed fluctuations were avoided.

Although an excessive half cone angle can make the material flow as an overall flow in the grain cylinder, the problems of a large hopper height and poor economy arise in the actual application process of grain cylinders. Therefore, it is necessary to comprehensively consider whether the material produces a severe segregation state, whether the retention time changes the nature of the material, and whether the unloading amount needs to be strictly controlled in the construction of grain cylinders. In the future, sound-absorbing materials and damping devices will be added to the inner edge of the grain cylinder to interfere with the transmission of velocity and acceleration between grain layers to reduce the occurrence of grain pulsation and avoid granary vibration.

This study focused on the effects of different storage height and different half cone angle on the periodic pulsation characteristics of a grain system during grain unloading and explored the interaction mechanism from the perspective of the particle layer system. Based on the periodic pulsation characteristics in the unloading stage explored in this paper, we will systematically study the effect of different half cone angles on the forces experienced by the plexiglass wall of the grain cylinder during unloading, explore the effect of particle layers with different heights in the periodic pulsation period on the forces experienced by the plexiglass wall of the grain cylinder during unloading, and further explore the mechanism of corn grain effect on granary from the perspective of mechanics. This study provides theoretical guidance for further analysis from the perspective of mechanics, and the subsequent mechanical research can be mutually verified with the results of this study. The two parts not only form a progressive relationship, but also form a complete system.

## 4. Conclusions

(1) An accurate discrete element model of corn grain was established by laser scanning and natural rest angle comparison. A model of the vertical grain cylinder was established according to similarity theory. The maximum error of the comparison between high-speed imaging and the discrete element simulation grain unloading test was 2.71%. The real grain unloading environment of a vertical grain cylinder could be simulated by the discrete element method.
(2) With the increase in grain unloading height, the average speed of corn grain increased in unit time. The higher the unloading height, the more significant the periodic pulsation characteristics. The grain pulsation in the grain cylinder was gradually transmitted upward, and the pulsation characteristics were gradually magnified in the process of transmission. However, the average speed decreased with increasing height.
(3) When the half cone angle was in the range of 30–65°, the speed of the top corn grain increased gradually with increasing half cone angle. The phenomenon of periodic pulsation became more significant with increasing half cone angle. The half cone angle in the range of 65–70° was the critical state of corn grain changing from funnel flow to overall flow in the grain cylinder.

## Figures and Tables

**Table 1:** Simulation parameters.

Parameters	Numerical Value	References
Density of corn grain/(kg/m^3^)	1150	[34]
Poisson’s ratio of corn grain	0.357	
Elastic modulus of corn grain/(MPa)	1500	
Plexiglass density/(kg/m^3^)	1180	[35]
Poisson’s ratio of plexiglass	0.5	
Elastic modulus of plexiglass/(MPa)	1770	
Static friction coefficient between corn grain and corn grain	0.275	[34,36]
Rolling friction coefficient between corn grain and corn grain	0.05	
Collision recovery coefficient between corn grain and corn grain	0.382	
Static friction coefficient between corn grain and plexiglass	0.351	[37]
Rolling friction coefficient between corn grain and plexiglass	0.093	
Collision recovery coefficient between corn grain and plexiglass	0.709	[36]

## Data Availability

All data are presented in this article in the form of figures and tables.
